# Risk factors of developing psychological problems among frontline healthcare professionals working in the COVID-19 pandemic era: a meta-analysis

**DOI:** 10.1186/s12889-023-16820-3

**Published:** 2023-10-12

**Authors:** Hongquan Wan, He Li, Shuxin Luan, Chunguo Zhang

**Affiliations:** 1 Department of Mental Health, the First Hospital of Jilin University, No.1 Xinmin Road, Changchun, 130021 China; 2 Department of Pain Medicine, the First Hospital of Jilin University, No.1 Xinmin Road, Changchun, 130021 China

**Keywords:** Mental health, Anxiety, Depression, Healthcare professionals, COVID-19 pandemic

## Abstract

**Background:**

This study sought to evaluate the risk factors behind developing psychological problems as per specific mental health assessment instruments. This study focuses specifically on frontline healthcare professionals of the COVID-19 pandemic era, and evaluated the psychological assessment of frontline healthcare professionals.

**Methods:**

Studies reporting on the psychological assessment of frontline healthcare professionals were retrieved from the PubMed, Embase, Web of Science, Ovid, EBSCO, and Cochrane Library databases. The recommended method was used to assess the risk of bias of the included studies. The random-effects method was applied when significant heterogeneity was observed.

**Results:**

The combined results from the 20 included articles indicated that frontline healthcare professionals had a higher risk of developing anxiety in comparison with non-frontline healthcare workers, with similar levels of depression scoring were observed. Healthcare providers aged > 40 years had a lower probability of developing anxiety and seemed to experience minimal depression. Conversely, frontline workers had a higher incidence of anxiety than that of depression. Being single (not in a relationship) could influence the PHQ-9 scores instead of those concerning the GAD-7. The gender gap was not proven to be significantly wide between healthcare professionals with or without anxiety; however, being male was proven to be positively correlated with depression.

**Conclusion:**

In general, the risk factors for susceptibility to psychological problems among frontline healthcare professionals during the COVID-19 pandemic concerned those of a lower age, being single, being male, and being engage in frontline healthcare work.

**Supplementary Information:**

The online version contains supplementary material available at 10.1186/s12889-023-16820-3.

## Introduction

The outbreak of the novel coronavirus disease 2019 (COVID-19) has not only imposed great threats to people’s physical health but — given its severity, rapid spread, and global influence — also causes tremendous agony. The World Health Organization announced that the COVID-19 pandemic is a public health emergency of international concern [1], with the virus having had a direct impact on the health of millions of people worldwide. In addition, the pandemic and virus pose a significant threat to mental health globally [2–4]. Healthcare professionals, including medical staff and affiliated healthcare workers, are on the frontline of the battlefield to stand against the pestilence. As an unprecedented global challenge, supporting the mental health of healthcare professionals is of great importance. Therefore, studies are needed to specify the psychological effects on medical staff and for addressing some of the organizational, team-based, and individual concerns for the pragmatic support of staff during this pandemic. Leaders at all levels of healthcare organizations will find this to be a valuable resource. In confronting the COVID-19 pandemic, healthcare staff may experience different types of stress (such as grief, moral injury, or guilt) and reactions (such as acute stress reactions, coping, fear, anxiety, depression, burnout prevention, and post-traumatic stress disorder) [5, 6].

A meta-analysis has reported that the estimated prevalence of anxiety, depression, and insomnia were 23.2%, 22.8%, and 38.9%, respectively, among healthcare workers during the COVID-19 pandemic [7]. Another meta-analysis conducted to analyze the psychological impact of COVID-19 on the general population reported that the prevalence of anxiety and depression was 33% and 28%, respectively, and that the prevalence of anxiety and depression was highest among patients with pre-existing conditions and COVID-19 infections (56% and 55%) [8]. Other synthetic studies have reached a consensus regarding the viewpoint that the COVID-19 pandemic has increased the prevalence of mental health issues among the global population, and particularly among healthcare workers, chronic disease patients (non-COVID-19 patients), COVID-19 patients, and persons being quarantined [9–15]. Different levels — ranging from the organizational provision of psychological support to mutual help among the work team — could make a difference in the maintenance of mental health and could encourage medical staff to be more optimistic [9–15]. In addition, a machine-learning-based study has suggested that a team leader could protect their staff from psychological crises through positive communication with others, which includes advice from experts in mental well-being, as well as those with direct experiences from the frontline of the pandemic [6]. Consideration of the psychological integrity of healthcare workers is also important. The overall findings mentioned above indicate that the mental health of frontline healthcare workers requires more attention and that there is a need to focus on necessary prevention and intervention methods. Reactive policies to manage the rapid spread of COVID-19 have had wide-ranging effects on the social and economic burden faced by populations worldwide. Psychiatrists and psychologists, for example, can play a vital role in understanding COVID-19 related mental trauma. Kisely et al. [16] argues that clear communication, access to adequate personal protection, adequate rest, and the affordance of both practical and psychological support are effective interventions for mitigating psychological distress. Additionally, Pollock et al. suggest that workplace interventions — such as training, structure, and communication —, psychological support interventions — such as counselling and psychological services — and multifaceted interventions could be selected as useful interventions that are beneficial to the resilience and mental health of frontline workers [11].

However, with the emergence of various clinical studies concerning the incidence of mental problems and psychological issues among frontline healthcare workers during the COVID-19 pandemic, a summary these data would facilitate in obtaining mixed information and in identifying the risk factors related to mental problems. This could help us take precise countermeasures by targeting specific psychological issues for improving the mental health conditions of healthcare professionals [17]. In this study, and based on accumulated evidence, we have aimed to clarify the risk factors for the susceptibility to psychological problems among frontline healthcare professionals during the COVID-19 pandemic. Accordingly, an suggestion for global frontline medical staff was made based on a comprehensive analysis. As such, this study’s findings may help maintain the psychological well-being of frontline medical staff.

### Methods

#### Search strategy

This study was conducted in accordance with the Preferred Reporting Items for Systematic Reviews and Meta-Analyses (PRISMA) guideline [18]. Web-based electronic databases (PubMed, Embase, Web of Science, Ovid, EBSCO, and the Cochrane Library) were searched by two authors (Hong-Quan Wan and He Li) and covering the period prior to February 1, 2022. The retrieval fields included: “mental health,” “psychiatry,” “psychological intervention,” “quality of life,” “healthcare professionals,” “medical staff,” “caregiver,” “COVID-19,” “clinical study,” and “clinical trial.” In addition, the reference lists of the included articles were retrieved in case anyone fitting the criteria was omitted via the inclusion criteria. The search strategies included different combinations of search terms; for instance: “(mental health OR psychia*, psycholog* OR mental) AND quality of life AND (healthcare profession* OR medical staff OR caregiver) AND COVID-19.”

### Study selection and data extraction

The Medical Subject Headings field was used to search the online databases. Keywords such as “mental health,” “anxiety,” “depression,” “healthcare professionals,” and “COVID-19 pandemic,” among others, were searched in the electronic database as subject terms. Quality of life (QoL) was measured using the Professional Quality of Life: Compassion Satisfaction and Fatigue Version 5 (ProQOL) scale, which is a reliable tool for measuring “burnout,” “secondary traumatic stress,” and “compassion satisfaction” among professional medical staff [19–21]. The Patient Health Questionnaire-9 (PHQ9) [22, 23] was used to evaluate depression. The Seven-item Generalized Anxiety Disorder Scale (GAD-7) [24, 25], a practical self-report anxiety questionnaire that proved valid in primary care, was applied to measure anxiety conditions, as previously reported. Two researchers independently searched the databases and screened the results according to inclusion and exclusion criteria. An additional investigator, acting as a referee, was invited to provide a final judgement in cases of a divergence in opinions. The inclusion criteria were as follows — study design: cohort study with retrospective or prospective design; population: healthcare professionals including doctors, nurses, and any other caregivers during the COVID-19 pandemic; and measurement tools: assessment of mental health condition; ProQOL, PHQ9, and GAD-7 were applied as psychological measurement tools. A frontline workplace was defined as a COVID-19 designated hospital with an isolation ward. The exclusion criteria were as follows: studies without proper comparison groups, repetitive studies, reviews, case reports or case series, editorials, or letters to the editor. The Newcastle-Ottawa Scale (NOS) was used to assess the quality of the studies, and the results are shown in Supplementary Table 1.

### Data synthesis and analysis

Noteexpress Bibliography Software (version 3.2.0; Beijing Aegean Software Co., Ltd., China) was used to create reference citations and for scrutinizing duplicate records. The corresponding data from each study, including the name of the first author, year of publication, country, sample size, baseline demographic characteristics, and outcome measures, were extracted and collected by two independent investigators.

### Statistics

Stata statistical software (version 12.0, University of Texas Stata Company) was used to analyze and integrate the extracted data. If original data was shown as the form of quantile value: the median (50th percentile), 25th percentile and 75th percentile, was transferred to a mean ± SD format via a previously published method [26–28]. Statistical heterogeneity of the extracted data was evaluated using the Inverse Variance (I-V) formula. The I^2^ statistic was used to describe heterogeneity within the study. Studies with *I*^*2*^> 50% or a *P* value < 0.05 were considered to have high heterogeneity. The random-effects model was used to calculate the aggregate estimates; otherwise, when low heterogeneity was found among the included studies, the fixed-effects model was applied. The weighted mean difference (WMD) was calculated for the continuous variables. Binary variables are expressed as odds ratios (OR) and a by a 95% confidence interval (95% CI). Publication bias was evaluated using Egger’s test and presented as a funnel plot. A two-tailed *P*-value of < 0.05 was considered statistically significant. Sensitivity analysis was performed to evaluate the results obtained using the random-effects model.

## Results

### Database searching and study inclusion

**Fig. 1:**
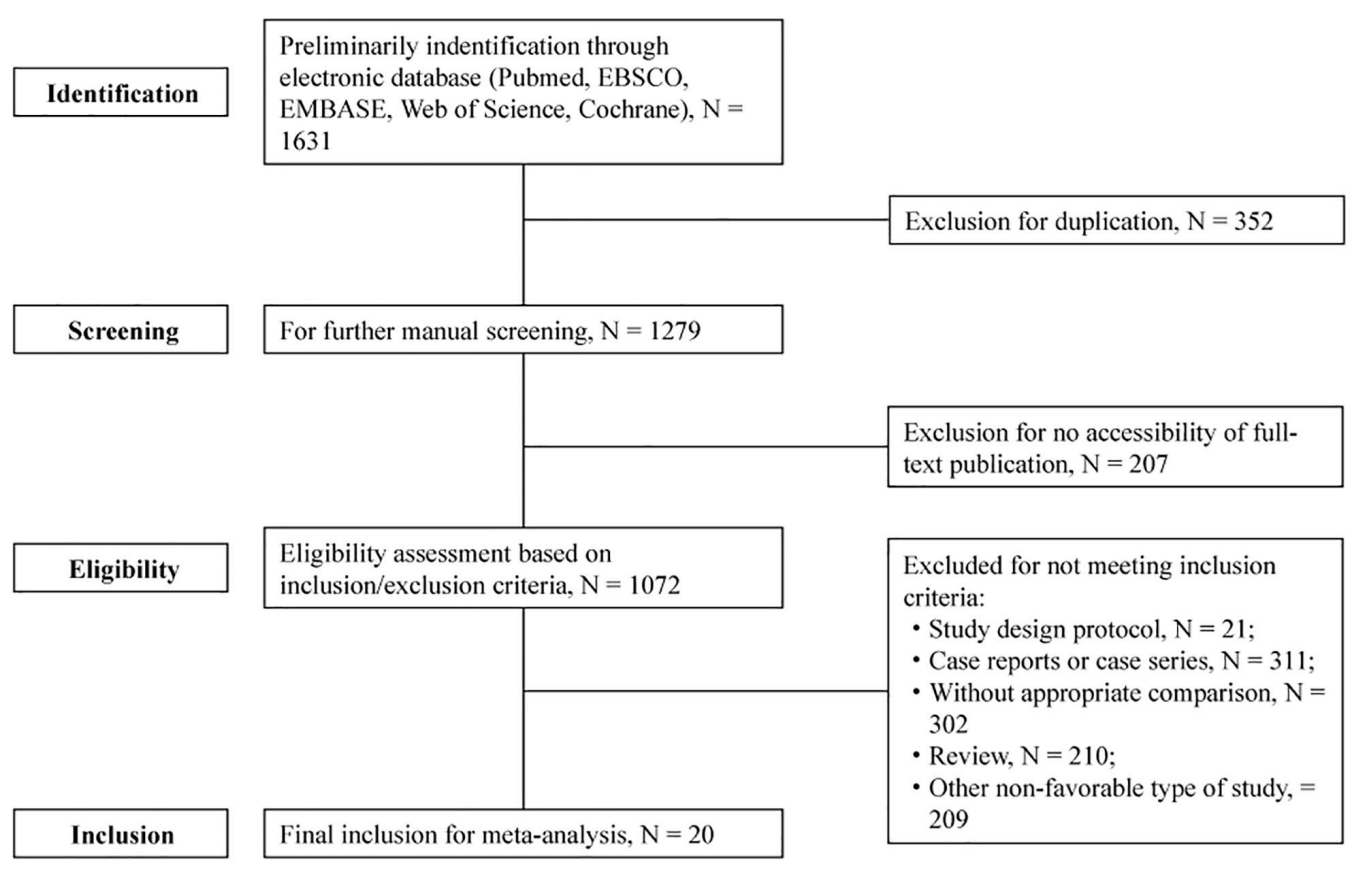
Literature retrieval and screening

Based on our retrieval strategy, 1,631 articles were identified, with 352 duplicates excluded. A total of 207 articles were further excluded because full-text versions were not accessible. After filtering by the inclusion/exclusion criteria, 20 articles [29–47] were finally included for further meta-analysis. A flow chart of the publication filtration is shown in Fig. 1. The characteristics of the included studies are shown in Table 1. All included studies were cross-sectional, and the patients’ demographic characteristics, at the baseline, are described in Table 2. A quality assessment of the included studies is presented in Supplemental Table 1.

**Table 1:** The characteristics of included studies

First Author	Year	Age (year)	Male (%)	Number	Nurses	Doctors	Healthcare assistants
Suryavanshi	2020	33.8 (7.5)	96 (49)	197	47 (24%)	124 (63%)	26 (13%)
Stojanov	2020	39.1 (7.3)	41 (34.4)	118	71 (59.8%)	47 (40.2%)	0
Trumello	2020	40.55 (11.49)	125 (19.9)	627		Unclassified	
Young	2020	NG	353 (24)	1,685	1,293 (76%)		392 (24%)
Huang	2020	40 (14.8)	334 (11.2)	1,747	1,413 (47.6%)	1,557 (52.4%)	
Manh	2020	31.4 (6.7)	55 (31.8)	173	109 (63%)	43 (24.9%)	21 (12.1%)
Vafaei	2020	NG	70 (30)	240	120 (50%)	70 (30%)	50 (20%)
Nathiya	2020	30 (14)	136(32.5)	418	191(45.7%)	135(32.3%)	92 (22%)
Khanna	2021	44.7 (12.1)	1,332 (56.7)	2,350		470	
Wańkowicz	2020	40.47 (4.93)	211 (47.8)	441		Unclassified	
Lasalvia	2020	44.4 (14.1)	539 (27.4)	2186	783 (35.7%)	667 (30.3%)	745 (34%)
Wang	2020	37.4 (7.7)	32 (22.4)	143	89 (62.2%)	54 (37.8%)	0
Zhang	2020	38.3 (8.0)	33 (70.2)	47	17 (36.2%)	22 (46.8%)	8 (17.0%)
Chatzittofis	2021	38.8 (11.4)	176 (42)	424	103 (24.3%)	178 (42%)	143 (33.7%)
Chen	2021	42 (5.7)	382 (42.5)	898		Unclassified	
Antonijevic	2021	40.38 (10.32)	135 (21.6)	625	364 (58.2%)	261 (41.8%)	0
Awano	2020	37 (3)	213 (25.1)	848	461 (45.0%)	104 (25.3%)	283 (29.7%)
Conti	2020	NG	219 (23.4)	933	933 (100%)	0	0
Wanigasooriya	2020	41.6 (14.1)	524 (19.9)	2,706		Unclassified	
Altmayer	2020	33.9 (7.8)	54 (78)	69	40 (58%)	11 (16%)	18 (26%)

NG: not given

**Table 2:** The patients’ demography at baseline

First Author	Year	Region	number of patients	Study design	Type of occupation	Follow-up duration
Suryavanshi	2020	India	197	cross-sectional	nurses, physicians, and other healthcare workers	2 weeks
Stojanov	2020	Serbia	118	cross-sectional	nurses and physicians	3 week
Trumello	2020	Italy	627	cross-sectional	nurses, physicians, and other healthcare workers	3 weeks
Young	2020	United States	1,685	cross-sectional	nurses and physicians	1 week
Huang	2020	China	1,747	cross-sectional	nurses and physicians	1 week
Manh	2020	Vietnam	173	cross-sectional	nurses, physicians, and other healthcare workers	4 weeks
Vafaei	2020	Iran	240	cross-sectional	nurses, physicians, and other healthcare workers	2 weeks
Nathiya	2020	India	418	cross-sectional	nurses, physicians, and other healthcare workers	2 weeks
Khanna	2021	India	2,350	cross-sectional	nurses, physicians, and other healthcare workers	1 week
Wańkowicz	2020	Poland	441	cross-sectional	nurses, physicians, and other healthcare workers	2 weeks
Lasalvia	2020	Italy	2,186	cross-sectional	nurses, physicians, and other healthcare workers	3 weeks
Wang	2020	China	143	cross-sectional	nurses and physicians	1 week
Zhang	2020	South Sudan	47	cross-sectional	nurses, physicians, and other healthcare workers	3 weeks
Chatzittofis	2021	Sweden	424	cross-sectional	nurses, physicians, and other healthcare workers	3 weeks
Chen	2021	China	898	cross-sectional	nurses, physicians, and other healthcare workers	8 weeks
Antonijevic	2021	Serbia	625	cross-sectional	nurses and physicians	1 week
Awano	2020	Japan	848	cross-sectional	nurses, physicians, and other healthcare workers	4 weeks
Conti	2020	Italy	933	cross-sectional	nurses	4 weeks
Wanigasooriya	2020	UK	2,706	cross-sectional	nurses, physicians, and other healthcare workers	8 weeks
Altmayer	2020	France	69	cross-sectional	nurses, physicians, and other healthcare workers	8 weeks

### Comparison of the psychological scale measurement between frontline healthcare professionals and non-frontline healthcare professionals against Sars-Cov-2

**Fig. 2:**
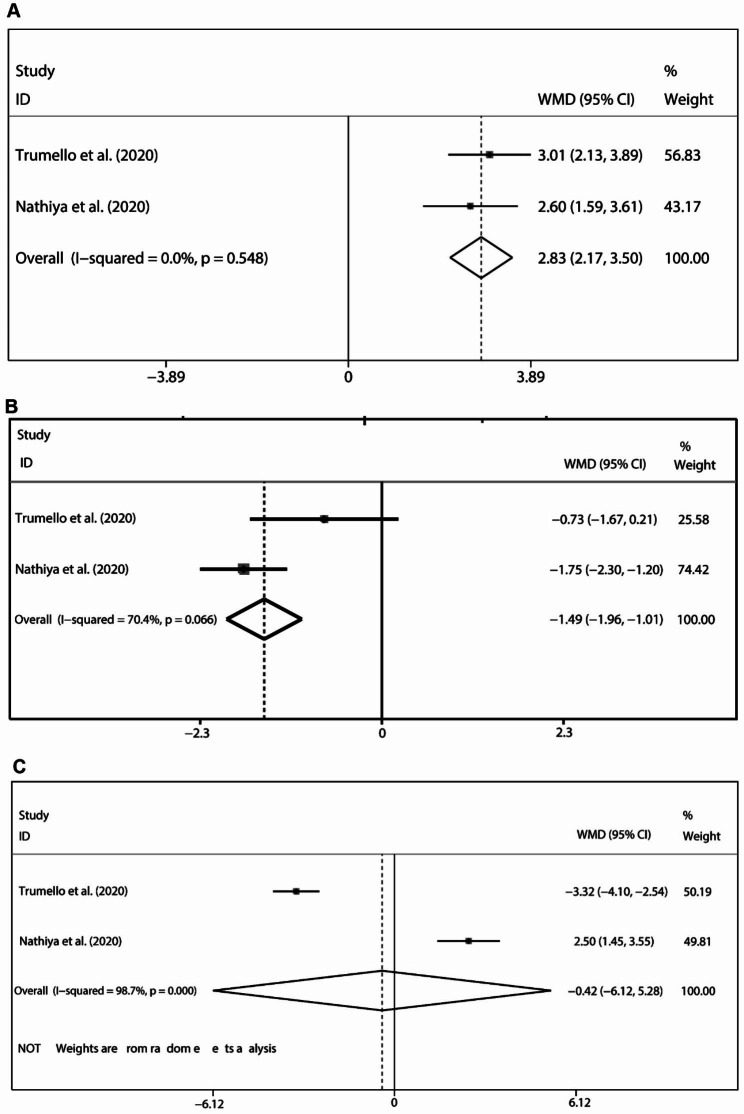
Comparison of the ProQOL-5 scale measurements between frontline healthcare professionals and non-frontline healthcare professionals against Sars-Cov-2: **(A)** results of the CS subscale; **(B)** results of the ST subscale; **(C)** results of the burnout subscale. ProQOL-5: Compassion Satisfaction and Fatigue Version 5; CS: compassion satisfaction; and ST: secondary traumatic stress

**Fig. 3:**
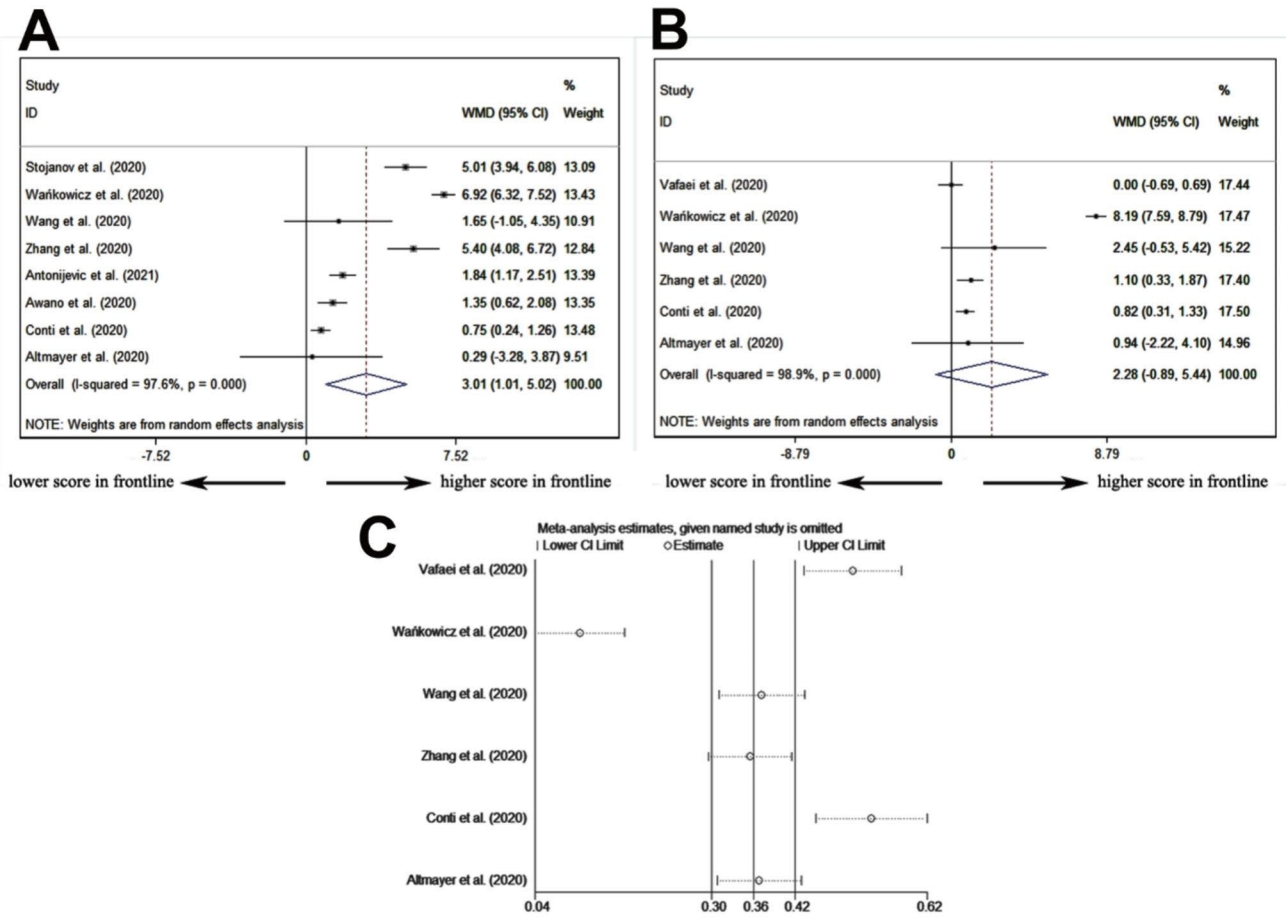
Comparison of the GAD-7 and PHQ-9 scale measurements between frontline healthcare professionals and non-frontline healthcare professionals against Sars-Cov-2: **(A)** result of the GAD-7 scale; **(B)** result of the PHQ-9 scale; **(C)** heterogeneity detection via sensitivity analysis. GAD-7: The seven-item Generalized Anxiety Disorder Scale; PHQ-9: Patient Health Questionnaire-9

WMD was calculated to reflect the difference in the pooled mean of the ProQOL-5 subscale over three dimensions: compassion satisfaction (CS), burnout, and secondary traumatic stress (ST) between frontline and non-frontline healthcare professionals. From two studies [31, 36], the CS subscale validated that frontline healthcare professionals acquired lower level scores (pooled WMD = -1.49, 95% CI: -1.96 – -1.01) without significant heterogeneity (*P* = 0.066) in term of compassion satisfaction, indicating lower levels of self-satisfaction experienced by frontline healthcare professionals (Fig. 2A). From the two same studies [31, 36], the ST subscale verified that frontline healthcare professionals attained higher scores (pooled WMD = 2.83, 95% CI:2.17–3.50) without significant heterogeneity (*I*^*2*^ = 0.0% and *P* = 0.548) in term of and secondary traumatic stress items (Fig. 2B). This revealed that frontline healthcare professionals were more susceptible to Post-Traumatic Stress Disorder (PTSD). When it came to the burnout subscale [31, 36], scores between frontline healthcare professionals and non-frontline healthcare professionals showed no difference (pooled WMD = -0.42, 95% CI: -6.15–5.28), and this manifested the professional burnout and professional belief (Fig. 2C). The GAD-7 was used to assess the anxiety and mood conditions of healthcare professionals. From eight studies [30, 38, 40, 41, 44–46, 48] in which the GAD-7 scale was referred to, healthcare professionals in frontline work places attained higher scores (pooled WMD = -1.49, 95% CI: -1.96 – -1.01), suggesting that being directly confronted with confirmed COVID-19 patients correlates with a greater risk of anxiety (Fig. 3A). However, because significant heterogeneity was detected (*I*^*2*^ = 70.4% and *P* < 0.001), sensitivity analysis was applied, and the heterogeneity (Figure sensi-WMD-GAD-7) was attributed to two studies [38, 46].

To examine the differential performance of the PHQ9 — a nine-item depression scale — as a screening and diagnostic instrument for assessing depression between frontline healthcare professionals and non-frontline healthcare professionals were reported in six studies [35, 38, 40, 41, 46, 48];, the pooled data did not attain a significant level of difference (pooled WMD = 2.28, 95% CI: -0.89–5.44), indicating similar levels of depression conditions (Fig. 3B). However, the data were characterized by significant heterogeneity (*I*^*2*^ = 98.9% and *P* < 0.001). In the heterogeneity analysis, one study [35, 38, 40, 41, 46, 48] contributed the most to the origin of heterogeneity (Fig. 3C). From the measurement results of the GAD-7 and PHQ-9 scales, frontline healthcare professionals were found to be more susceptible to anxiety than to depression.

### Risk factors of healthcare professionals susceptible to anxiety in the era of the Sars-Cov-2 (COVID-19) pandemic

**Fig. 4:**
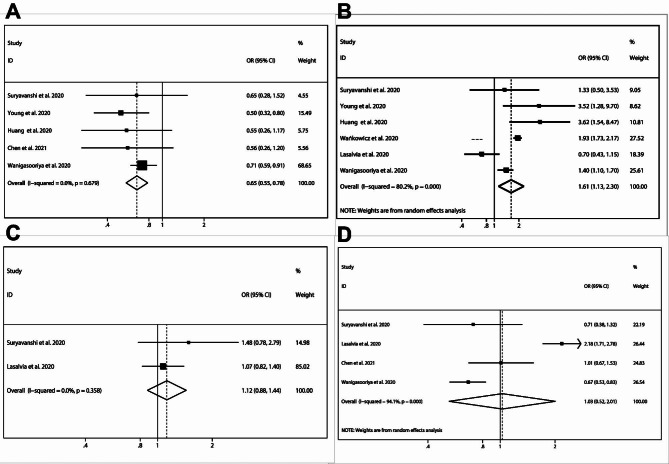
Risk factors of healthcare professionals susceptible to anxiety: **(A)** age > 40 years; **(B)** frontline place of work; **(C)** single marital status; **(D)** and male gender

Based on the data accessibility of the included studies, the related risk factors were divided into four dimensions: age > 40 years, frontline workplace (designated hospital/isolation ward), single marital status, and male sex. These factors were investigated to determine whether they independently influenced mental health. In six included studies [29, 32, 33, 38, 39, 47], anxiety was evaluated using the GAD-7 scale, and healthcare professionals were divided into anxiety and non-anxiety groups based on the questionnaire measurements. In five included studies [29, 32, 33, 39, 47], healthcare workers older than 40 years of age had a lower probability of developing anxiety (pooled OR = 0.65, 95% CI:0.55–0.78), with no heterogeneity being detected (*I*^*2*^ = 0.0% and *P* < 0.679) in the pooled data (Fig. 4A). In comparison with healthcare professionals in non-frontline work places (designated hospital/isolation ward) — six studies [29, 32, 33, 38, 39, 47] — frontline workers had a higher incidence of anxiety (pooled OR = 0.65, 95% CI:0.55–0.78) by random effects model (*I*^*2*^ = 80.2% and *P* < 0.001), indicating that they were predominantly suffering from anxiety (Fig. 4B). Only two studies [29, 39] reported the influence of marital status on the results of the GAD-7 measurements. Based on limited data, being single did not influence the GAD-7 scores (pooled OR = 1.12, 95% CI:0.88–1.44), revealing that marital status does not seem to be related to anxiety among healthcare professionals (Fig. 4C). Nonetheless, in four included studies [29, 39, 43, 47], there was no difference in anxiety incidences between male and female workers (pooled OR = 1.03, 95% CI:0.52–2.01), suggesting that the gender gap in terms of this question was not proven to be significantly wide between healthcare professionals with or without anxiety (Fig. 4D).

### Risk factors of healthcare professionals susceptible to depression in the era of Sars-Cov-2 (COVID-19) pandemic

**Fig. 5:**
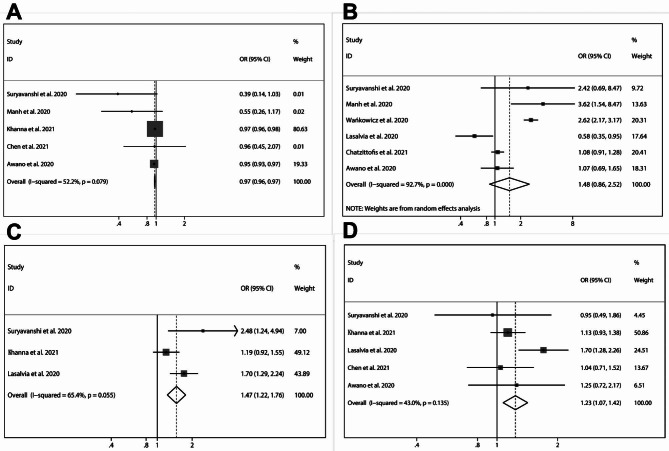
Risk factors of healthcare professionals susceptible to depression: **(A)** age > 40 years; **(B)** frontline place of work; **(C)** single marital status; **(D)** and male gender

In eight of the included studies [29, 34, 37, 39, 42, 43, 45, 47], depression was evaluated using the PHQ-9 scale, with healthcare professionals divided into depression and non-depression groups based on the measurements of the aforementioned questionnaire. In five of the included studies [29, 34, 38, 42], healthcare workers above the age of 40 seemed to suffer little from depression (pooled OR = 0.97, 95% CI:0.96–0.97), with no heterogeneity being detected (*I*^*2*^ = 52.5% and *P* = 0.079) in the pooled data (Fig. 5A). By a random effects model (*I*^*2*^ = 92.7% and *P* < 0.001), in comparison with healthcare professionals in non-frontline places of work, depression tended to be more developed among frontline workers (pooled OR = 1.48, 95% CI:0.86–2.52). However, in six studies [29, 34, 37, 43, 45, 47], no statistically-significant difference was detected, indicating that frontline workplaces potentially influence the mental condition of depression (Fig. 5B). Three studies [28, 36, 38] report the influence of marital status on the PHQ-9 questionnaire results. From pooled data with low heterogeneity (*I*^*2*^ = 65.4% and *P* = 0.055), being single could influence the PHQ-9 scores (pooled OR = 1.47, 95% CI:1.22–1.76), revealing that marital status was significantly related to depression among healthcare professionals (Fig. 5C). To our surprise, after integrating five included studies [29, 37, 39, 43, 45], being male proved to be positively correlated with depression (pooled OR = 1.23, 95% CI:1.07–1.42), suggesting that sex contributes to the development of depression (Fig. 5D).

### Sensitivity analysis and publication bias analysis

Sensitivity analysis in terms of frontline workplace on the GAD-7 and PHQ-9 scale measurements indicated that there was no origin of heterogeneity in the included studies (Supplemental Figure A and B). After removing any study individually, the heterogeneity did not increase, nor did the statistical significance change. Therefore, no studies needed to be excluded from the overall pooled analysis, and it was appropriate to use a random-effects model to process the extracted data.

In terms of publication bias analysis (Begg’s and Egger’s tests) verified a symmetrical distribution of included publications (Begg’s test: *P* = 0.902; Egger’s test: *P* = 0.825), suggesting that there was no publication bias among the included articles (Supplemental Figure C).

## Discussion

Twenty articles were included in the present meta-analysis. In terms of quality of life, frontline healthcare professionals were characterized by lower levels of self-satisfaction and higher levels of traumatic stress. However, there was no significant difference in professional burnout between groups. Direct confrontation with confirmed COVID-19 patients was correlated with a higher risk of anxiety; however, similar levels of depression scores were acquired between frontline and non-frontline healthcare professionals. Healthcare workers aged > 40 years have a lower probability of developing depression and anxiety. Frontline healthcare professionals predominantly suffer from anxiety, whereas frontline workplaces scarcely influence the mental condition of depression. Marital status was significantly related to depression among healthcare professionals; however, anxiety had little influence. Being male contributed to the development of depression rather than anxiety. Three dimensions of the ProQOL-5 scale, which reflect professional-related compassionate satisfaction and professional fatigue, were evaluated. The CS subscale validated that frontline healthcare professionals had lower scores, indicating a lower level of self-satisfaction among frontline healthcare professionals. The ST subscale verified that frontline healthcare professionals attained higher scores, indicating that they were the more susceptible to potential traumatic stress. Regarding the burnout subscale, scores between frontline and non-frontline healthcare professionals showed no difference, manifesting as professional burnout and professional belief. Direct confrontation with confirmed COVID-19 patients correlated with a higher risk of anxiety. However, similar depression scores were obtained between frontline and non-frontline healthcare professionals.

Healthcare workers aged > 40 years had a lower probability of developing anxiety, and seemed to experience minimal depression. Conversely, frontline workers had a higher incidence of anxiety than of depression. Being single (not in a relationship) could influence the PHQ-9 scores instead of the GAD-7 scores. The gender gap was not proven to be significantly wide between healthcare professionals with or without anxiety; however, being male was proven to be positively correlated with depression. In the present study, healthcare workers aged > 40 years had lower probabilities of developing depression and anxiety. Frontline healthcare professionals predominantly suffer from anxiety, whereas frontline workplaces scarcely influence the mental condition of depression. Marital status was significantly related to depression among healthcare professionals; however, anxiety had little influence. Being male contributed to the development of depression rather than anxiety.

Our study suggests that older workers have fewer anxiety and depression-related problems than younger workers. We concluded that being older not only meant more accumulated social experience, but a richer working experience as well, all of which could weaken the negative influence of the COVID-19 pandemic. Working on the frontline against COVID-19 could cause more anxiety, which originates from the concern or fear of being infected [49, 50]. However, differences in workplace did not seem to increase the risk of developing depression. Shader et al. [51] claimed that during the COVID-19 pandemic, depression was mostly attributed to unemployment, death, and isolation, among others. However, some of the factors that could demoralize someone — such as unemployment — scarcely existed among healthcare workers. In contrast, the outbreak of COVID-19 highlighted the importance of healthcare professionals, which might lift their spirits. Contrary to our conventional viewpoint, men were more vulnerable to depression than anxiety. A previously-published meta-analysis revealed that depression in males should not be overlooked in the general population [52]. Our study indicates that male healthcare providers may be more prone to depression. Workers who were single were also more likely to experience anxiety or depression. To some extent, marital status represents physical and psychological support from an intimate spouse. Marital status had been proven to be important in some special groups [53, 54].

Based on this meta-analysis, frontline healthcare professionals generally performed poorly on the QoL scale when compared to non-frontline healthcare professionals, indicating a more severely-impaired QoL for those directly in contact with COVID-19 patients. In addition, being over 40 years of age, working in a frontline place of work, being single, and being male are independent risk factors that could potentially predict whether a certain healthcare professional is susceptible to psychological problems during this extraordinary period. Early interventions with this special group could improve the mental health of such healthcare providers. Ongoing studies have focused on the psychological issues and intervention methods for frontline workers. One concerned a randomized control-designed clinical trial aimed at assessing the efficacy and acceptability of a brief online cognitive behavioral therapy program specifically developed for healthcare workers [55]. Another ongoing randomized control trial study would disclose whether participation in regular debriefings can prevent burnout among intensive care unit employees [56]. These studies would help optimize the strategy to tackle mental problems resulting from the COVID-19 pandemic among frontline healthcare professionals in this era.

### Limitations

The current study was not registered which might lead to some bias, however, this meta-analysis was done following the instruction of the PRISMA guideline to minimize the potential bias. Due to the limited research methods and study designs, the included studies were mainly characterized by a cross-sectional design. Therefore, a longer follow-up duration is needed to verify the accuracy of risk factors related to mental health. Additionally, multi-strategy-based interventions for improving the mental health of healthcare professionals during the COVID-19 pandemic should be considered in the future.

## Conclusion

In general, the risk factors of susceptibility to psychological problems among frontline healthcare professionals during the COVID-19 pandemic were being younger than 40 years of age, being single, being male, and working in a place considered to be on the frontline of healthcare work.

### Electronic supplementary material

Below is the link to the electronic supplementary material.

Supplementary Material 1

Supplementary Material 2

## Data Availability

The datasets used and/or analyzed in the current study are available from the corresponding author upon reasonable request.
