# Supplementary material for: Risk factors of developing psychological problems among frontline healthcare professionals working in the COVID-19 pandemic era: a meta-analysis
Source: BMC Public Health. 2023 Oct 12;23:1991. doi: 10.1186/s12889-023-16820-3 (PMC10571421; doi:10.1186/s12889-023-16820-3)
Supplement: Supplementary file 1 — Supplementary Material 1 [file 12889_2023_16820_MOESM1_ESM.docx]

**Supplemental Figure** Sensitivity analysis and publication bias analysis: sensitivity analysis in term of frontline work place in GAD-7 scale **(A)**and PHQ-9 scale **(B)** measurements analysis; publication bias by Begg’s and Egger’s methods **(C)**.

GAD-7: The 7-item Generalized Anxiety Disorder Scale; PHQ-9: Patient Health Questionnaire-9


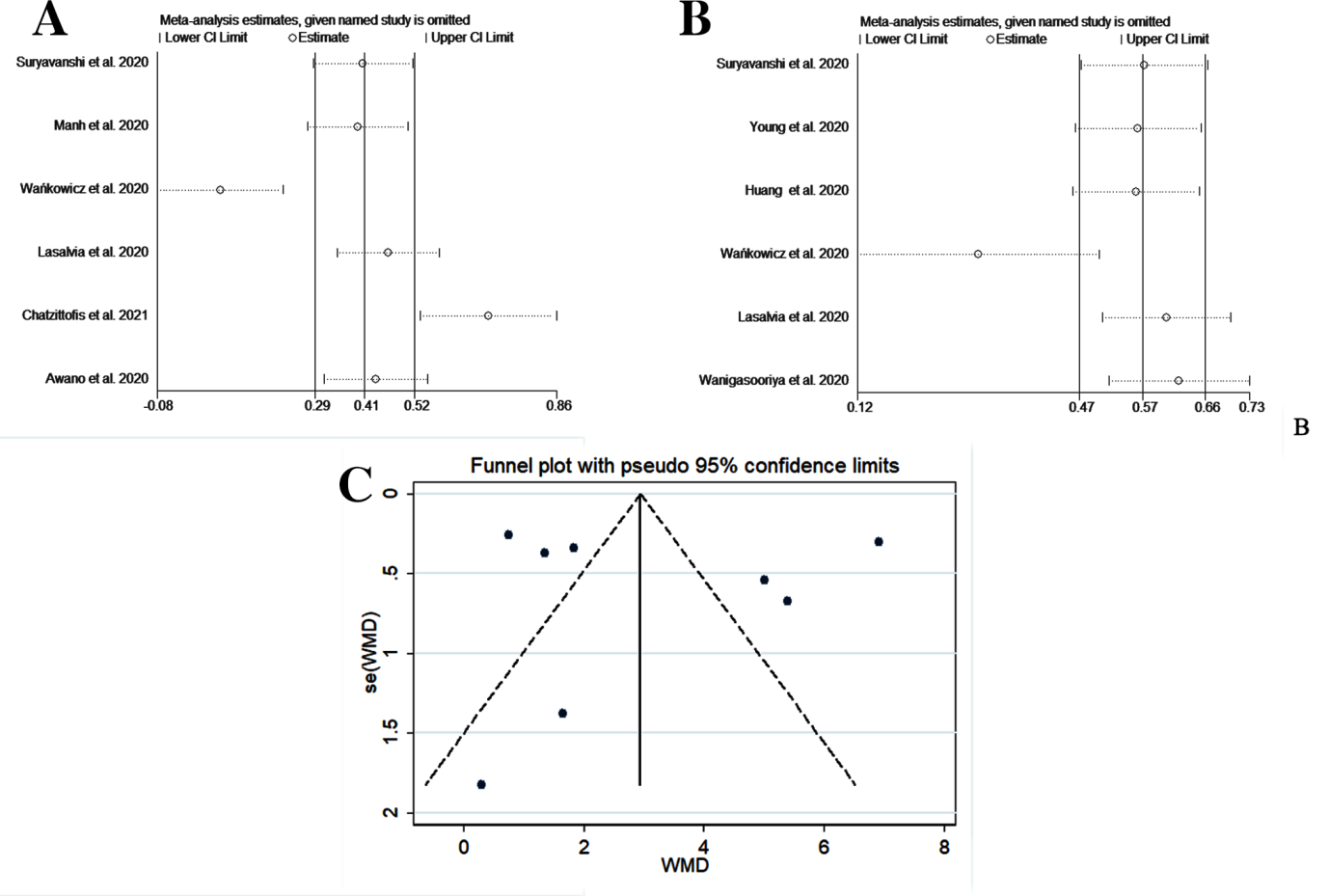


**Supplementary Table 1** Scale for assessing the quality of studies.
